# Assessment on the ownership and use of mosquito nets in Mozambique

**DOI:** 10.1590/S1518-8787.2016050006335

**Published:** 2016-11-24

**Authors:** Jorge Alexandre Harrison Arroz, Francisco Chirrute, Chandana Mendis, Marta Honesta Chande e, Veronique Kollhoff

**Affiliations:** I World Vision Mozambique. Malaria Project Global Funded. Maputo, Moçambique

**Keywords:** Mosquito Nets, supply & distribution, Mosquito Nets, utilization, Insect Bites and Stings, prevention & control, Health Knowledge, Attitudes, Practice, Cross-Sectional Studies

## Abstract

**OBJECTIVE:**

To assess the ownership and use of mosquito nets in 2014, in Mozambique.

**METHODS:**

This observational and cross-sectional study assessed, in February and March 2015, 69 districts (nine of 11 provinces of Mozambique) that have benefited from the mass distribution of mosquito nets. The Lot Quality Assurance Sampling methodology was used. Each locality was denominated supervision area. The Lot Quality Assurance Sampling opts for a minimum of 19 households (in this case, we decided for a minimum of 100 households per district) from each supervision area to assess an indicator (in this case, two indicators were assessed: ownership and use of mosquito nets). Two questions guided the research: a) received a mosquito net; b) used a mosquito net the night before.

**RESULTS:**

A total of 6,725 households were assessed. Eighty three percent of them had received mosquito nets in the campaign. Of the 6,232 respondents, 82.0% said they used mosquito nets the night before. The districts of the provinces with low coverage of ownership and use were Tete (69.5% and 60.0%, respectively), Zambezia (79.0% and 60.0%, respectively), and Gaza (81.6% and 70.7%, respectively). The largest coverage of ownership and use were observed in the districts of Nampula (96.7% and 93.8%, respectively) and Niassa (86.0% and 85.4% respectively).

**CONCLUSIONS:**

In the districts assessed, the progression of ownership and use of mosquito nets is satisfactory. Nampula and Niassa are the only provinces where ownership and use are at desired levels.

## RESUMO

**OBJECTIVO:**

Avaliar a posse e o uso das redes mosquiteiras no ano de 2014 em Moçambique.

**MÉTODOS:**

Este estudo observacional transversal avaliou, em fevereiro e março de 2015, 68 distritos (nove das 11 províncias de Moçambique) que se beneficiaram da distribuição de redes em massa. Usou-se a metodologia *Lot Quality Assurance Sampling*. Cada localidade foi designada de área de supervisão. O *Lot Quality Assurance Sampling* opta por um mínimo de 19 agregados familiares (neste caso decidiu-se um mínimo de 100 agregados familiares por distrito) de cada área de supervisão, a fim de avaliar um indicador (neste caso dois indicadores foram avaliados: posse e uso de redes mosquiteiras). Duas perguntas nortearam a pesquisa: a) recebeu rede; b) usou rede na noite anterior.

**RESULTADOS:**

Foram avaliados 6.725 agregados familiares . Desses, 83,0% tinham recebido redes na campanha. Dos 6.232 inqueridos, 82,0% disseram que usaram na noite anterior. As províncias com distritos com menores coberturas de posse e uso foram Tete (69,5% e 60,0%, respectivamente), Zambézia (79,0% e 60,0%, respectivamente) e Gaza (81,6% e 70,7%, respectivamente). As maiores coberturas de posse e uso foram observadas nos distritos de Nampula (96,7% e 93,8%, respectivamente) e Niassa (86,0% e 85,4%, respectivamente).

**CONCLUSÕES:**

Nos distritos avaliados, a progressão para a posse e uso de redes mosquiteiras é satisfatória. Nampula e Niassa são as únicas províncias onde a posse e o uso estão em níveis desejados.

## INTRODUCTION

Malaria is a major public health problem in the world, with about 207 million cases and 627 thousand deaths per year; most of the cases (80.0%) and deaths (90.0%) happen in Africa^a^.

In Mozambique, malaria is endemic and represents 45.0% of all the cases observed in outpatients and about 56.0% of hospitalizations in pediatric wards^b^. According to the last demographic health survey carried out in 2011, the prevalence of malaria in children from six to 59 months is 35.1%; the provinces of Zambezia and Nampula showed the highest prevalence (55.2% and 42.2%), while Maputo City and Maputo Province showed the lowest (2.5% and 4.8%)^c^.

The use of long-lasting insecticidal nets (hereinafter referred to as “insecticide-treated net”) may reduce morbidity and mortality caused by malaria, especially in children and pregnant women^2^ ^,^ ^3^.

In 2011, in Mozambique, half of the households had at least one insecticide-treated net, 35.0% of children under five years slept under an insecticide-treated net the night before, as well as 34.0% of the pregnant women^2^.

In 2012, the National Malaria Control Program (NMCP) distributed 2,560,216 insecticide-treated nets (935,997 in antenatal visits and 1,624,419 in universal coverage campaigns, hereinafter referred to as “campaign”^d^) throughout Mozambique. In 2013, 3,098,675 insecticide-treated nets were distributed (885,023 in antenatal visits and 2,213,652 in campaign). In 2014, 5,672,392 insecticide-treated nets were distributed (1,258,998 in antenatal visits and 4,413,404 in campaign)^e^.

Several factors influence the ownership and use of insecticide-treated nets: availability (access), behaviors, knowledge, durability of the nets (estimated at three years^6^ ^,^ ^f^), among others^4^ ^,^ ^6^ ^,^ ^a^. In addition to the availability (which the National Malaria Control Program ensures in antenatal visits and campaigns), communication activities for behavior change are conducted with messages about the correct and consistent use of insecticide-treated nets. However, inappropriate use is still reported^4^ ^,^ ^a^.

By the effort invested in providing insecticide-treated nets and in communication activities for behavior change, this study was conducted to assess the ownership and use of insecticide-treated nets to understand the outcomes of the several interventions from the point of view of the beneficiaries.

## METHODS

Mozambique is located on the East Coast of southern Africa, with a surface area of approximately 799,380 km^2^. It borders to the North with Tanzania, to the West with Malawi, Zambia, Zimbabwe, and South Africa, to the South with Swaziland and South Africa, and to the East with the Indian Ocean^g^.

An observational and cross-sectional study, of exploratory nature, was conducted in 68 districts (nine of the 11 provinces of Mozambique) that have benefited from the distribution of nets in campaign. The survey was conducted in February and March 2015.

The Lot Quality Assurance Sampling method was used. In each province the districts that would benefit from the 2012-2014 campaign were selected. Each district was denominated “supervision unit”. Each location (smallest geographical and administrative area in the district) was denominated “supervision area”. The LQAS opts for a minimum of 19 households or houses (the sample of 19, by the LQAS method, has a precision of 92.0% and provides an acceptable error level for decision-making in management) from each supervision area to assess ownership and use of insecticide-treated nets. The survey of at least 100 houses was considered in case the total of houses surveyed in the district did not reach at least 100 households. Two questions guided the research: a) received insecticide-treated nets in the campaign; b) used the insecticide-treated net the night before. The use of the nets was assessed with questions and by observation (i.e., if the insecticide-treated net was placed in the sleeping compartment). The study was approved by the National Committee of Bioethics for Health (Ref: 030_1/CNBS/2014).

## RESULTS

This study comprised 6,725 households (houses) in 68 districts (98.9% of houses assessed). Of these, 5,582 (83.0%) had received insecticide-treated nets in the campaign. Concerning the use of insecticide-treated nets the night before, of the 6,232 surveyed, 5,112 (82.0%) said (and we noted) they used it the night before. Regarding the ownership of nets, the assessed districts of Niassa, Nampula, Inhambane provinces achieved the campaign’s goal of 85.0% coverage. As for the use, the assessed districts of Niassa, Cabo Delgado, Nampula, and Manica provinces achieved the campaign’s goal of 85.0% coverage (Tables 1 and 2).

**Table 1:** Ownership and use of insecticide-treated nets by districts assessed per provinces. Mozambique, 2015.

Province	Question 1				Question 2			
	
	Ownership of insecticide-treated nets				Use of insecticide-treated nets			
	
	Yes	%	No	%	Yes	%	No	%
Gaza	653	81.6b	147	18.4	564	70.7c	234	29.3
Cabo Delgado	1,401	82.3^b^	302	17.7	1,249	89.2^a^	152	10.8
Sofala	814	81.5^b^	185	18.5	804	80.5^b^	195	19.5
Zambezia	395	79.0^c^	105	21.0	369	73.8^c^	131	26.2
Inhambane	728	86.7^a^	112	13.3	683	81.3^b^	157	18.7
Niassa	338	86.0^a^	55	14.0	316	85.4^a^	54	14.6
Tete	139	69.5^c^	61	30.5	120	60.0^c^	80	40.0
Manica	737	81.9^b^	163	18.1	644	87.4^a^	93	12.6
Nampula	377	96.7^a^	13	3.3	363	93.8^a^	24	6.2
Mozambique	5,582	83.0^b^	1,143	17.0	5,112	82.0^b^	1,120	18.0

^a^ Desirable Situation (coverage ≥ 85.0%).

^b^ Intermediate situation (coverage between 80.0% and 85.0%).

^c^ Undesirable Situation (coverage < 80.0%).

**Table 2:** Ownership and use of insecticide-treated nets per provinces, and progress toward the campaign’s goal. Mozambique, 2015.

Province	Ownership (%)	Use (%)	% of districts covered	Goal “Ownership” (%)	Goal “Use” (%)	Difference “ownership” and goal	Difference “use” and goal	Progression toward the “use” goal
Niassa	86.0	85.4	66.7	85.0	85.0	+1.0	+0.4	Desirable situation^a^
Cabo Delgado	82.3	89.2	100	85.0	85.0	-2.7	+4.2	Desirable situation^a^
Nampula	96.7	93.8	23.5	85.0	85.0	+11.7	+8.8	Desirable situation^a^
Zambezia	79.0	73.8	38.5	85.0	85.0	-6.0	-11.2%	Undesirable situation^c^
Tete	69.5	60.0	22.2	85.0	85.0	-15.5	-25.0	Undesirable situation^c^
Manica	81.9	87.4	90.0	85.0	85.0	-3.1	+2.4	Desirable situation^a^
Sofala	81.5	80.5	100	85.0	85.0	-3.5	-4.5	Intermediate situation^b^
Inhambane	86.7	81.3	90.0	85.0	85.0	+1.7	-3.7	Intermediate situation^b^
Gaza	81.6	70.7	100	85.0	85.0	-3.4	-14.3	Undesirable situation^c^
Mozambique	83.0	82.0	68.0	85.0	85.0	-2.0	-3.0	Intermediate situation^b^

^a^ No difference toward the goal or positive value of the difference.

^b^ Difference toward the goal and negative value between -1 and -10.

^c^ Difference toward the goal and negative value between > -10.

## DISCUSSION

To verify the validity of this assessment on the ownership and use of insecticide-treated nets, we used the NetCalc software, version 2.0, to estimate the ownership of insecticide-treated nets in 2014. We used the 2007 Census^h^ statistical data to fill the variables of the NetCalc menu: population (20,632,434), average of people per household (six), and percentage of the annual population growth (2.9%). The percentage of the population at risk of getting malaria is 100%. To estimate the current net coverage, statistical data from the demographic health survey of 2011^b^ were used: percentage of households with any mosquito net (57.0%), percentage of households with any insecticide-treated net (51.4%), and percentage of insecticide-treated nets (50.2%). The durability considered for insecticide-treated nets was three years. Data on the distribution of the nets in the period after the demographic health survey of 2011, i.e., from 2012 to 2014, were obtained from the National Malaria Control Program and were presented in the introduction.

The results of NetCalc, version 2.0, showed that, in 11 provinces (150 districts) of Mozambique, the ownership of insecticide-treated nets was 79.1%, which is close of the 83.0% found in this survey conducted in nine provinces and 68 districts.

A study carried out in Sofala Province, Mozambique, in 2010 and 2011, showed ownership coverage of 98.0% and 93.0%, one and 14 months after the campaign, respectively. The proportion of sleeping spaces with an insecticide-treated net hung was 61.0% and 65.0% in the first and fourteenth month after the campaign, respectively^8^.

These results validate this study’s assessment on ownership and shows that Mozambique took an important step by providing insecticide-treated nets for the population. In addition, the communication strategies used for behavior change and the campaign associated to the continuous distribution of insecticide-treated nets in antenatal visits have been showing the desired effect.

Studies have shown that the lack of access is the main limiting factor for people at risk of getting malaria not to protect themselves^a^. A comparison between the proportion of the population with access to insecticide-treated nets and the proportion that sleeps under them suggests that high percentages (86.0%) of the population with access to these nets use them, which indicates that the efforts made to encourage the use of insecticide-treated nets have been positive. In Manhiça, southern Mozambique, most (62.5%) pregnant women prefer insecticide-treated nets over other interventions for malaria prevention^1^.

However, scenarios of non-usage or of inappropriate use are reported, mainly in coastal regions or near lakes and streams, where the basis of survival is fishing, and insecticide-treated nets are used for this purpose^4^ ^,^ ^5^ ^,^ ^7^. The economic pressure has an impact on preventive measures^9^; thus, it is probable that as long as a relief of this pressure is not registered (greater food security and lower prevalence of hunger), the use of insecticide-treated nets for fishing will continue.

This research is a guide for the implementation of several strategies. In general, in the districts assessed, the progression to the ownership and use of the insecticide-treated net is satisfactory compared with the desired goal. Tete, Zambezia, and Gaza provinces have precarious districts. Nampula and Niassa are the only provinces where ownership and use are at desired levels. Nampula, Cabo Delgado, Niassa, and Manica are the only provinces where the use of insecticide-treated nets is at desired levels.

In general, all districts must reinforce efforts in communication activities for behavior change, to increase the sense of ownership and use of insecticide-treated net, especially for the districts that have coverage of ownership and use below 85.0%. The National Malaria Control Program must continue to endorse the availability of insecticide-treated nets with campaigns and by using a system of continuous distribution (antenatal visit, expanded program on immunization, schools, or other), ensuring the maintenance and strengthening of communication strategies for behavior change to achieve and promote universal access.

## Footnotes

a WHO - Global Malaria Programme. World malaria report. Geneva; 2013. Available from: http://www.who.int/malaria/media/world_malaria_report_2013/en/

b República de Moçambique, Ministério da Saúde, Direcção Nacional de Saúde Pública. Plano Estratégico da Malária 2012-2016. Maputo; 2012. Available from: http://www.rollbackmalaria.org/files/files/countries/mozambique2012-2016.pdf

c República de Moçambique, Ministério da Saúde, Instituto Nacional de Estatística; MEASURE DHS/ICF International. Inquérito demográfico e de saúde 2011. Maputo, MZ; 2011. Available from: http://www.dhsprogram.com/pubs/pdf/FR266/FR266.pdf

d In Mozambique, the goal is 85.0%.

e Programa Nacional de Controlo da Malária (PNCM). Relatórios anuais 2012, 2013 e 2014. Maputo. MISAU.

f Roll Back Malaria Vector Control Working Group’s Continuous LLIN Distribution Systems Work Stream. Continuous long-lasting insecticidal net distributions: a guide to concepts and planning. Geneva: Roll Back Malaria Partnership Secretariat; 2011. Available from: http://www.rollbackmalaria.org/files/files/partnership/wg/wg_itn/docs/ws3/3-Guide_to_continuous_distribution_strategy.pdf

g Ministério da Saúde (MZ), Direcção Nacional de Saúde Pública. Programa Nacional de Controlo da Malária: Plano Estratégico da Malária 2012-2016. Maputo; 2012 [cited 2015 Sept 10]. Available from: http://www.rollbackmalaria.org/files/files/countries/mozambique2012-2016.pdf

h Instituto Nacional de Estatística. III Recenseamento Geral da População e Habitação 2007. Moçambique; 2007 [cited 2015 Feb 8]. Available from: http://www.ine.gov.mz/estatisticas/estatisticas-demograficas -e-indicadores-sociais/projeccoes-da-populacao/indicadores-demograficos-mocambique-2007-2040.xlsx/view> and <http://www.ine.gov.mz/operacoes-estatisticas/censos/censo-2007/rgph-2007

## References

[1] Boene H, González R, Valá A, Rupérez M, Velasco C, Machevo S (2014). Perceptions of malaria in pregnancy and acceptability of preventive interventions among Mozambican pregnant women: implications for effectiveness of malaria control in pregnancy. PLoS ONE.

[2] Gamble CL, Ekwaru JP, Kuile FO (2009). Insecticide-treated nets for preventing malaria in pregnancy. Cochrane Database Syst Rev..

[3] Lengeler C (2004). Insecticide-treated bed nets and curtains for preventing malaria. Cochrane Database Syst Rev.

[4] McLean KA, Byanaku A, Kubikonse A, Tshowe V, Katensi S, Lehman AG (2014). Fishing with bed nets on Lake Tanganyika: a randomized survey. Malar J.

[5] Minakawa N, Dida G, Sonye GO, Futami K, Kaneko S (2008). Unforeseen i of bed nets in fishing villages along Lake Victoria. Malar J.

[6] Morgan J, Abílio AP, Pondja MR, Marrenjo D, Luciano J, Fernandes G (2015). Physical durability of two types of long-lasting Insecticidal nets (LLINs) three years after a Mass LLIN distribution campaign in Mozambique, 2008-2011. Am J Trop Med Hyg.

[7] Mwina H, Mutelekesha A, Hakizimana G, Katonda K, Ebaka D, Ntahuga L (2011). The Strategic Action Program for the Protection of Biodiversity and Sustainable Management of Natural Resources in Lake Tanganyika and its Basin.

[8] Plucinski MM, Chicuecue S, Macete E, Colborn J, Yoon SS, Kachur SP (2014). Evaluation of a universal coverage bed net distribution campaign in four districts in Sofala Province, Mozambique. Malar J.

[9] Stratton L, O’Neill MS, Kruk ME, Bell ML (2008). The persistent problem of malaria: addressing the fundamental causes of a global killer. Soc Sci Med.

